# Epidemiology of Hepatitis A Virus Infections, Germany, 2007–2008

**DOI:** 10.3201/eid1511.090214

**Published:** 2009-11

**Authors:** Mirko S. Faber, Klaus Stark, Susanne C. Behnke, Eckart Schreier, Christina Frank

**Affiliations:** Robert Koch Institute, Berlin, Germany

**Keywords:** Molecular epidemiology, hepatitis A virus, Germany, phylogeny, risk factors, travel, migration, surveillance, viruses, research

## Abstract

Communicating vaccination recommendations may help reduce infections.

Approximately 60% of hepatitis A virus infections in Germany occur in persons without a travel history to disease-endemic areas and for whom sources of infection are unknown. Recommendation of pretravel vaccination fails to prevent the remaining imported infections. Using enhanced surveillance in 2007–2008, we analyzed epidemiologic patterns of hepatitis A in Germany and appropriateness and adequacy of current immunization recommendations. Young patients with a migration background who had visited friends and family in their ancestral countries accounted for most imported cases. Phylogenetic analysis showed high diversity of sequence data and clustering of strains with similar regions of origin or patient migration backgrounds. Virologic findings are compatible with those of low-incidence countries, where virtually all infections are directly or indirectly imported from other regions. Germans with a migration background are seen as a special risk group so far insufficiently reached by pretravel vaccination advice.

After decades of steady decreases (*1*), annual cases of hepatitis A in Germany in 2005 and 2006 remained at a relatively constant level (≈1,200 reported cases, incidence 1.5/100,000 population). The actual number of infections estimated on the basis of prevalence of immunoglobulin (Ig) G against hepatitis A virus (HAV) in Germany is presumed to be higher. Mild or subclinical infections, especially in children, are not detected by surveillance (*2*). A seasonal pattern is observed every year; most reported cases occur in late summer and fall.

In Sweden, notification data indicate that most cases are imported (*3*). However, ≈60% of reported cases in Germany occur in persons who deny recent travel to disease-endemic areas (*4*), similar to the situation in France (*5*). Exact sources and risk factors for these autochthonous infections in Germany often remain unknown because routine surveillance data lack detail.

Molecular markers, such as nucleotide sequence patterns, have proven useful for elucidating modes and chains of transmission or identifying new risk groups and factors. These factors would otherwise be difficult to determine because of the long incubation period (15–50 days) for hepatitis A and often unapparent connections between persons involved in outbreaks (*6*).

A 1-year study was initiated by the Robert Koch Institute (RKI) to characterize and compare imported and nonimported HAV infections in Germany and determine HAV genotype distribution and sources of infection. We assessed whether the virus is endemic among certain (unrecognized) risk groups, whether (and which) imported infections play a role in secondary autochthonous infections, and which population groups should be targeted for specific prevention approaches (e.g., immunization).

## Methods

### Routine Epidemiologic Data

The study was conducted from the 14th calendar week of 2007 through the 13th calendar week of 2008. Acute hepatitis A has been a notifiable disease in Germany for many years. Since 2001, laboratories have reported infections (test results indicative of acute HAV infection, detection of specific IgM in serum, or detection of HAV RNA in serum or feces by PCR) to local health departments. These departments collect information on case-patients (age, sex, travel history, date of onset, clinical symptoms, duration of hospitalization), take preventive measures to avoid further spread (including recommending vaccination of contacts, barring infected food handlers from working), and report standard case information electronically to state health authorities and RKI in a form in which names and addresses are removed. Data can be grouped on all levels to indicate outbreaks.

### Additional Epidemiologic Data

To transcend standard information obtained for each reported infection and add a virologic perspective, we collected additional case information on all HAV infections in Germany over a 1-year period. The 16 state health departments in Germany were requested to participate and coordinate distribution and collection of questionnaires to all local public health departments. These departments recorded additional case information obtained during routine case investigations on paper forms (additional case information sheets). Data included details of travel, concurrent health conditions, and potential migration background. These investigations consisted of telephone interviews with patients (rarely with their physicians as proxies). Completed forms were sent to RKI without names and addresses but did contain case codes. Accommodations other than hotels were defined as those presumably involving closer contact with the local population or exposure to food prepared under potentially suboptimal hygienic conditions (e.g., private accommodations, hostels, or campgrounds).

Persons with a migration background were defined as those who moved to Germany after 1949 as non-German nationals, children born in Germany to non-German nationals, or children born to at least 1 parent belonging to either of these groups. Adults were defined as persons >18 years of age.

### Laboratory Data

We obtained serum samples from >10% of persons with HAV infections diagnosed in Germany during the study. To facilitate sequencing and phylogenetic analysis of a representative selection of HAV strains causing infections in Germany during the study, we asked >120 large private laboratories, university clinics, and hospitals in Germany to provide serum samples for patients for whom IgM against HAV was detected at their facilities. Samples were either sent to RKI immediately or stored at –20°C and sent in larger batches.

### Collation of Data Sources

Information recorded on paper forms was entered into a database and matched to electronically transmitted routine data by case codes. All symptomatic cases reported from participating states were analyzed. Clinical specimens were matched to questionnaire and surveillance data according to anonymous patient information (year and month of birth, sex, crude area of residence, date of blood sampling) provided by the laboratories.

### Isolation and Sequencing of HAV RNA

HAV RNA was isolated from serum samples by using a Viral RNA Mini Kit (QIAGEN, Hilden, Germany). Reverse transcription and first-round amplification of the capsid protein (VP1)/2A junction region of HAV were performed by using a Onestep RT-PCR Kit (QIAGEN) and primers (Table 1).

**Table 1:** Primers used for detection of HAV RNA by nested RT-PCR in clinical specimens from patients in Germany, 2007–2008*

Primer	Sequence (5′ → 3′)	Orientation, position, and use
HAV6a	ggA AAT ATT CAg ATT Agg YTg CCT Tgg T	Sense, 2793–2820, reverse transcription and first-round PCR
HAV6b	ggg AAC ATT CAg ATY AgA TTg CCW Tgg T	
HAV17a	CAA AgC TCT AgT RTC AgC AgT AAT TCC	Antisense, 3300–3326, reverse transcription and first-round PCR
HAV17b	CAA AgC CCT AgT RTC AgC AgT CAC TCC	
HAV8a	CTT TTg gAT TKg TTT CYA TTC AgA TTg C	Sense, 2882–2908, nested PCR and sequencing
HAV7a	gAA AAC TTC ATT ATT TCA TgM TCY TCW gT	Antisense, 3264–3292, nested PCR and sequencing

*HAV, hepatitis A virus; RT-PCR, reverse transcription–PCR. Positions in the HAV genome are given according to strain HM-175 (GenBank accession no. M14707).

Nested PCR was performed by using the HotStarTaq Master Mix Kit (QIAGEN). Purified products of the nested PCR (forward and reverse strands) were sequenced by using a 3130x ABI Prism Genetic Analyzer and a BigDye Terminator version 3.1 Cycle Sequencing Kit (PE Applied Biosystems, Weiterstadt, Germany).

### Statistical Analysis

Sequences were processed by using Lasergene SeqMan Pro software (DNASTAR, Inc., Madison, WI, USA), aligned by using the ClustalW algorithm (*7*), and optimized manually. Phylogenetic trees were constructed by using all available HAV sequences from obtained HAV IgM–positive samples (including HAV sequences from cases not reported). Sequence statistical and phylogenetic analyses were conducted by using MEGA4 (*8*). Sequences obtained are referenced in GenBank under accession nos. EU416232–EU416273 and EU825848–EU825918. For statistical analysis, we used Microsoft Excel 2003 (Microsoft, Redmond, WA, USA), SPSS version 15.0 (SPSS Inc., Chicago, IL, USA), and STATA version 10.1 (StataCorp, College Station, TX, USA).

## Results

### Study Population

A total of 1,213 HAV infections were reported. Among them, 952 (78.5%) were in patients with clinical symptoms consistent with acute hepatitis A. Of the 16 states in Germany, 13 participated in the intensified surveillance and contributed 1,037 (85.5%) of all reported infections and 816 (86%) of all reported symptomatic cases (maximal denominator for analysis). Additional case information sheets were available for 571 (70%) symptomatic cases. Serum samples positive for IgM against HAV were available for 189 (23.2%) cases; 95 (11.6%) were PCR positive.

A total of 74.6% of the cases were reported as single cases; the remainder were in recognized clusters. Among case-patients, 47.1% were male (Table 2); age range <1 to 90 years (median 32 years). Among nonadults, 81.1% were reported to have had jaundice. Among adults, the proportion of persons with jaundice decreased with age to 40.6% in persons >60 years of age.

**Table 2:** Characteristics of 816 patients tested for hepatitis A virus infection, Germany, 2007–2008

Characteristic	No. (%) patients	No. (%) patients with additional case information	No. (%) patients with serum samples available
Sex			
M	384 (47.1)	266 (46.7)	101 (53.7)
F	431 (52.9)	304 (53.3)	87 (46.3)
Age, y			
<1–9	168 (20.6)	136 (23.9)	44 (23.3)
10–19	138 (16.9)	107 (18.8)	32 (16.9)
20–39	157 (19.3)	119 (20.9)	43 (22.8)
40–59	180 (22.1)	116 (20.4)	39 (20.6)
>60	172 (21.1)	92 (16.1)	31 (16.4)
Hospitalized			
Yes	377 (46.4)	266 (46.6)	87 (46.3)
No	436 (53.6)	305 (53.4)	101 (53.7)
Imported infection			
Yes	346 (43.6)	269 (47.1)	89 (47.8)
No	447 (56.4)	302 (52.9)	97 (52.2)

Of case-patients, 46.4% were hospitalized for a median of 6 days (range 1–28 days). Among those with jaundice, no clear trend for age was found in 50.2% who were hospitalized. Among those without jaundice, the proportion of those hospitalized increased among persons >60 years of age (56.5%) when compared with that of younger persons (31.3%, relative risk [RR] 1.81, 95% confidence interval [CI] 1.35–2.41). Among those employed, absences from work ranged from 2 to 32 work days (median 6 days).

Overall, 43.6% of the infections were imported (i.e., infection acquired while traveling outside Germany). Nonadults (60.6% imported; p<0.001) and male patients (48.1% imported; p = 0.018) were overrepresented.

A migration background was reported by 42.2% of case-patients (78.8% of nonadults and 19.1% of adults), of whom 64.8% had been born in Germany. Among migration background, Turkey was reported most frequently (48.5%), followed by the former Yugoslavia (11.9%), southern and Southeast Asia (9.7%), and the former USSR (8.8%). Nonadults with a migration background lived with a larger number of household members (range 1–11 persons in addition to the case-patient, median 4) than those without a migration background (range 1–6 persons, median 3). Among adult case-patients, 5.2% were professional food handlers.

### Imported Infections and Comparison with Autochthonous Infections

Among known destinations, Turkey (35.6%) was reported most frequently, followed by the former Yugoslavia, Egypt, and Spain (Table 3). Most affected case-patients (63.9%), especially nonadults (82%), had not traveled for vacation or business but had visited friends or family. Median duration of travel preceding infection was 29 days (range 1–180 days). Accommodations other than hotels (e.g., staying with friends or family) predominated (73.1%), especially among those visiting Turkey (91.9%) and the former Yugoslavia (94.1%). A total of 92.9% of those infected in Turkey and 76.2% of those infected in countries of the former Yugoslavia had matching migration backgrounds; only 3 (16.7%) of those infected in Egypt had matching migration backgrounds. Of case-patients with migration backgrounds who had become infected in these ancestral countries, 75.9% from Turkey, 87.5% from the former Yugoslavia, and 100% from Egypt were nonadults.

**Table 3:** Travel characteristics of 346 patients infected with hepatitis A virus, Germany, 2007–2008

Characteristic	No. (%) patients
Destination	
Turkey	89 (35.6)
Former Yugoslavia	24 (9.6)
Egypt	18 (7.2)
Spain	15 (6.0)
Pakistan	10 (4.0)
Morocco	7 (2.8)
Others	86 (34.5)
All	249 (99.7)
Duration of travel, d	
1–14	51 (22.7)
15–29	62 (27.6)
30–180	112 (49.8)
All	225 (100)
Type of travel	
Visiting friends or family	149 (63.9)
Other vacation	79 (33.9)
Business	5 (2.1)
All	233 (100)
Type of accommodation	
Private	141 (73.1)
Hotel or cruise ship	52 (26.9)
All	193 (100)

**Figure 1:**
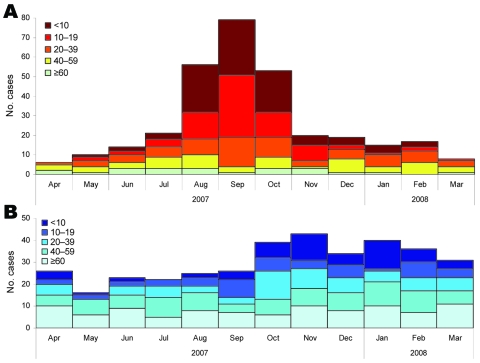
Reported cases of hepatitis virus A infection (n = 679) by month of onset and patient age group (y), Germany, 2007–2008. A) Imported cases. B) Nonimported cases.

Imported infections were most likely to cause disease from August through October, and a prolonged wave of autochthonous infections then followed from October through March (Figure 1). Among imported infections, children and persons <20 years of age were overrepresented (70.2% from August through October vs. 33.9% in other months; p>0.001). Among autochthonous cases, incidence in children and persons <20 years of age increased 62% from September through February compared with March through August. Autochthonous cases in adults >40 years of age were almost evenly spread throughout the year.

**Figure 2:**
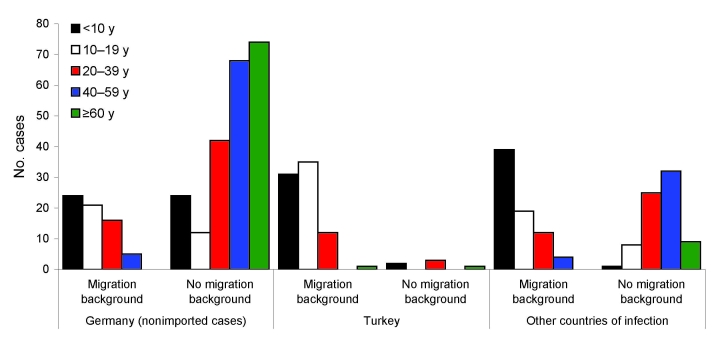
Age distribution (y) of persons with hepatitis A virus (HAV) infection by migration background and country where HAV infection was acquired (n = 520 with all 3 factors known), Germany, 2007–2008.

Case-patients with autochthonous infections were older than case-patients with imported infections and less likely to have migration backgrounds (Figure 2). Almost all patients >60 years of age had autochthonous infections.

A migration background was more likely among persons with imported infections; 52.8% of case-patients had a background of migration from Turkey. However, 23% of persons with autochthonous infections had a migration background (Table 4). Patients with autochthonous infections were older; 56.4% persons with nonimported cases were >40 years of age and 31.1% were >60 years of age, compared with only 24% and 7.5%, respectively, of persons of the same ages with cases of imported infections. Case-patients with imported infections were also slightly more likely to be male and part of case clusters than were autochthonous case-patients.

**Table 4:** Characteristics of patients with imported and nonimported hepatitis A virus infections, Germany, 2007–2008*

Characteristic	No. (%) patients with imported infections	No. (%) patients with nonimported infections	Total no. (%) infected patients	RR (95% CI)
Migration background	161 (63.9)	66 (23.0)	227 (42.1)	2.43 (2.01–2.95)
Male sex	180 (52.0)	194 (43.5)	374 (47.2)	1.21 (1.04–1.42)
Age <18 y	169 (48.8)	110 (24.7)	279 (35.2)	1.76 (1.51–2.04)
Age >60 y	26 (7.5)	140 (31.4)	166 (21.0)	0.31 (0.21–0.44)
Part of case cluster	79 (29.5)	65 (21.8)	144 (25.4)	1.22 (1.00–1.49)
<18 y of age with migration background	119 (91.5)	45 (57.7)	164 (78.8)	2.90 (1.73–4.88)

*RR, relative risk; CI, confidence interval.

Close contacts of 58.4% of the patients received prophylactic vaccinations (range 1–150 persons, median 3). Contacts were more frequently vaccinated in response to cases in nonadults (RR 1.594, 95% CI 1.374–1.849).

No large outbreaks were reported during the study. In the 13 participating states, 9 clusters with >5 infections were detected. The largest (13 symptomatic and 2 asymptomatic infections) was in a school with multiple generations of infection. The ultimate source of infection could not be elucidated. The school index case-patient had not traveled. However, the implicated HAV strain was genetically similar to strains from Turkey. In 4 of the 8 outbreaks with 5–7 infected persons, >1 travel-associated index cases were recognized, leading to 1–4 secondary infections in Germany.

### Detection of HAV RNA

HAV RNA was detected in 95 (50.3%) of 189 samples matching a reported case of symptomatic HAV infection. The likelihood of detecting HAV RNA in serum depended on clinical characteristics of patients (Table 5). Frequency of RNA detection increased with number of symptoms reported (p<0.02) and was negatively associated with patient age (p<0.001). Although this frequency was similar for nonadults and adults <39 years of age (mean 65%), RNA was detected in only 10% of symptomatic patients >60 years of age. No correlation was seen between duration from symptom onset to day of blood sampling (maximum 29 days) and positive results for HAV RNA.

**Table 5:** Serum samples positive for hepatitis A virus RNA by patient and disease characteristics for reported cases with symptomatic infections, Germany, 2007–2008

Characteristic	No. samples, n = 189	No. (%) positive samples, n = 95
Patient age, y		
<1–9	44	30 (68)
10–19	32	20 (63)
20–39	43	28 (65)
40–59	39	14 (36)
>60	31	3 (10)
Sex		
M	102	55 (54)
F	87	40 (46)
Signs and symptoms		
Abdominal pain	83	40 (48)
Fever	73	45 (62)
Jaundice	115	69 (60)
Increased transaminase levels	83	39 (47)
No. above items reported		
1	53	27 (51)
2	98	43 (44)
3	27	20 (74)
4	6	5 (83)
Probable place of infection		
Germany	115	49 (43)
Abroad	69	44 (64)

### Molecular Epidemiology

**Figure 3:**
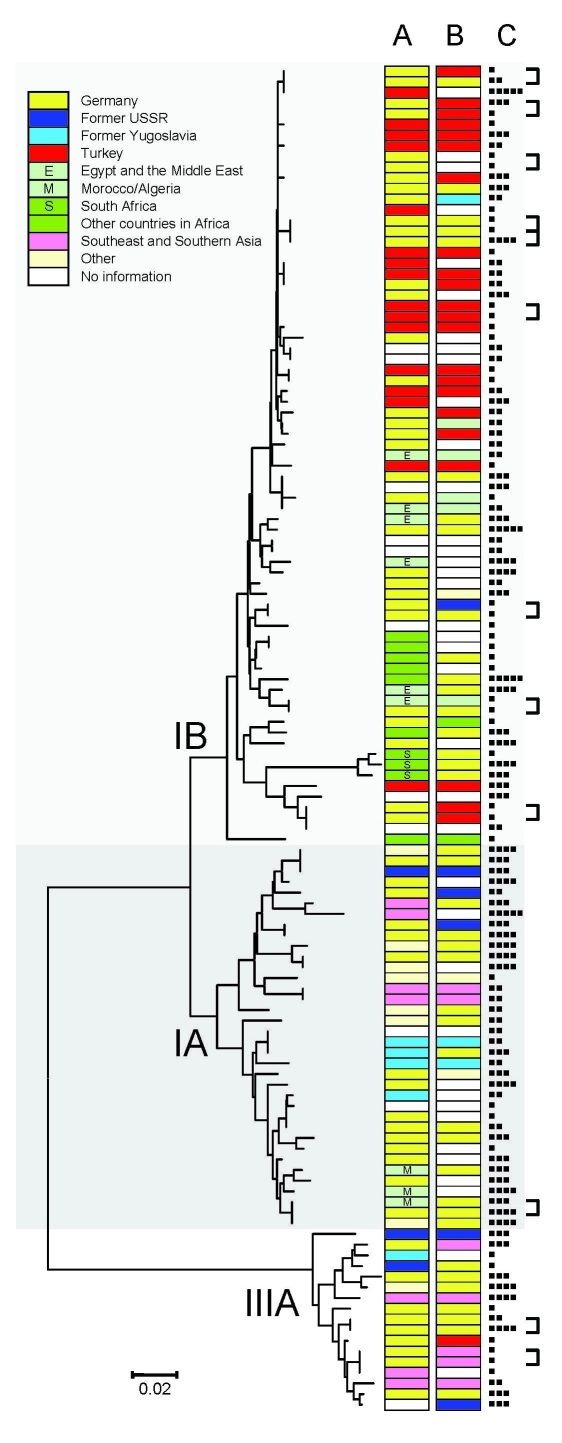
Neighbor-joining phylogenetic tree of a 348-bp section of the viral capsid protein 1//2A junction region of hepatitis A virus (HAV) constructed by using the Kimura 2-parameter distance model. Place of infection (A), migration background (B), and age of case-patients (■, 0–9 y; ■■, 10–19 y; ■■■, 20–39 y; ■■■■, 40–59 y; ■■■■■, >60 y) (C) are shown for each HAV isolate. Linked cases as judged by health departments are indicated by brackets. HAV subgenotypes are indicated by roman numerals and letters. Scale bar indicates nucleotide substitutions per site.

A PCR-generated 348-bp fragment was available for analysis of isolates with detectable HAV RNA from 126 patients. Of these patients, 73 (57.9%) had genotype IB strains, 36 (28.6%) had genotype IA strains, and 17 (18.6%) had genotype IIIA strains. Sequences differed in <8.9%, <7.5%, and <4.9% of positions within genotype IB, IA, and IIIA strains, respectively. Sequence variability was reflected in the diversity of countries or regions from which sequences originated (Figure 3).

Phylogenetic analysis showed that imported strains clustered according to region or country. Imported genotype IB strains were isolated from patients who traveled to Turkey, the Middle East, and Africa. Strains acquired in South Africa formed a small but distinct subcluster. Patients infected with genotype IA strains reported traveling to eastern Europe, Asia, South America, and northwestern Africa. The small number of imported genotype IIIA strains was obtained from patients who had traveled to eastern and southeastern Europe and Central and southern Asia. Three strains imported from Spain (2 genotype 1A and 1 genotype 3A) were genetically more distant from each other than to strains from other regions (e.g., northern Africa).

Strains from patients with known migration backgrounds (Figure 3) but no travel histories clustered with imported strains of the respective region. This finding was evident in a clade containing 35 highly related genotype IB strains, of which 14 were imported from Turkey. Of 23 persons with a background of migration from Turkey and for whom sequence data were available, 18 (78%) were infected with strains that were found within this clade. Furthermore, among persons who traveled to Turkey and available information on possible migration background (n = 12), all also had a background of migration from Turkey. Strains isolated from patients with autochthonous infections but without known migration backgrounds were nearly as genetically diverse as imported strains. Individual autochthonous strains, however, were frequently very homogeneous to individual imported strains.

Most (53.2%) HAV sequences obtained were found only once. Another 18.3% were isolated from smaller outbreaks of hepatitis A, which had already been detected through routine surveillance (Figure 3). Remaining sequences (28.6%) were each seen 2 or 3 times without any known epidemiologic connection. The exception was a strain found in 12 patients who represented sporadic cases and smaller outbreaks. This strain is part of the clade from Turkey mentioned earlier; among autochthonous cases with this strain, no clustering of time or place of infection was apparent.

## Discussion

Detailed epidemiologic data for 70% of symptomatic cases reported in participating states during the study (60% of all cases) enabled us to characterize incident cases of HAV infection in Germany better than using routine surveillance data alone. Although deaths from hepatitis A are rare, frequent hospitalization and work time missed by patients or adults caring for sick children emphasize the need to focus on hepatitis A.

Despite existing vaccination recommendations for travelers to countries with high or intermediate levels of endemicity for hepatitis A, >40% of cases were directly related to travel. Among these cases, persons with any migration background were overrepresented, especially those who had a background of migration from Turkey. The German Statistical Office in 2006 reported that 16% of adults and 26% of children (overall 19%) living in Germany had a migration background, compared with 63.9% of our patients with imported cases. Among those with a migration background who lived in Germany, 22% were from Turkey (*9*), but 48.5% of the case-patients had a Turkish migration background.

Among persons with infections imported from Turkey, 92% had a migration background even though Turkey is also a popular travel destination for persons from Germany without a migration background. These findings are consistent with surveillance data from Denmark, which show that 80% of travel-associated hepatitis A cases during 2002–2006 occurred in immigrants or children of immigrants, of whom 78% had acquired the disease while visiting their ancestral country (*10*).

Persons who travel to visit friends and family abroad are at greater risk for many preventable infectious diseases than persons traveling for other purposes, such as tourism (*11*). This higher risk for patients with migration backgrounds may be associated with regions visited. For example, persons who live on the Aegean coast of Turkey have lower prevalences of antibodies against HAV, which is indicative of the current level of HAV circulation, than do persons who live in eastern Anatolia, where many immigrant families have their roots (*12*).

Travelers who had stayed at private lodgings, visited friends or family, and traveled longer appeared to be overrepresented among cases. This result is supported by several studies, which demonstrated that persons planning visits to friends and relatives are less likely than other travelers to be vaccinated against HAV (*13*–*15*). Low vaccination coverage may be caused by lack of awareness by patients and physicians that when visiting friends and family in a hepatitis-endemic area, a vaccination before travel is recommended. Older persons with migration backgrounds may be immune to HAV because of previous exposure during childhood in their country of origin, but younger persons with migration backgrounds require vaccinations to acquire immunity. Persons with migration backgrounds, similar to friends and relatives they are visiting, may not be aware of hepatitis A because in many areas to which this disease is endemic, most infections occur in early childhood, and hepatitis A is rarely detected. Lower food safety standards for home cooking and contact with children in whom the virus circulates may facilitate acquisition of HAV by travelers when they stay with friends and family.

Population groups with a higher risk for acquiring HAV infection abroad are also more likely to acquire secondary infections. Molecular and questionnaire data showed that geographic origin of HAV strains most often matched the origin of patients with imported and autochthonous cases. These findings indicate that cases are imported by persons who visit home countries and that at least limited autochthonous spread of cases occurs among close contacts. Children of migrants born and raised in countries with low incidences of hepatitis A and who have no previous exposure and immunity to HAV can facilitate introduction of HAV into large households and the general community through schools or childcare facilities (e.g., outbreaks in Denmark [*16*] and the Netherlands [*17*]). In the Netherlands, hepatitis A immunization campaigns specific for children traveling to hepatitis A–endemic areas have proven to be useful for reducing the incidence of HAV infections among persons with migration backgrounds and others (*18*). Using vaccination to protect those at risk for primary infections while abroad would also preclude secondary spread in Germany.

Phylogenetic analysis of HAV sequences obtained showed a greater diversity of strains than that reported in a similar study in Amsterdam, the Netherlands (*19*). The 3 major genotypes were found in Germany. Within these genotypes, only limited relatedness but many unique strains were observed. As described in another study, HAV strains clustered according to geographic origin. This pattern is compatible with patterns from countries with a low incidence of HAV, where no or limited transmission occurs outside risk groups and most infections are caused by importation from areas outside the countries (*20*).

The largest cluster of strains observed was that of several highly related genotype IB strains. This cluster included only strains from cases imported from Turkey or nonimported cases from patients with a background of migration from Turkey. These strains were obtained throughout Germany during several months. Thus, relatedness may reflect endemicity to Turkey rather than endemic spread among a specific population in Germany.

Lack of distinct clusters containing predominantly autochthonous cases suggests that supraregional, unrecognized outbreaks did not occur during the study. Although infections secondary to imported cases may be frequent, especially in the immediate vicinity (household, family) of a case-patient, infection chains quickly terminate. This finding is also likely to result in part from satisfactory hygienic conditions in Germany but is also likely a result of effective tracing and vaccination of case-contacts by local health departments. Although the exception, larger outbreaks in daycare centers (*21*), those caused by food products (*22*), and those among men who have sex with men (*23*) are likely to be detected through routine surveillance. The largest outbreak detected in Germany in recent years involved tourists who had stayed at the same hotel in Egypt and was caused by consumption of contaminated orange juice (*24*).

The results of our study also provide information on specificity of surveillance data. Especially among older hospitalized patients, frequently without jaundice, IgM-positive serum samples were mostly negative for HAV RNA, which suggests false-positive serologic results. HAV IgM–positive samples that showed negative results by reverse transcription–PCR probably showed false-positive HAV IgM results for patients with persisting HAV IgM (*25*), cross-reactions in the test (e.g., in acute-phase infections with Epstein-Barr virus [*26*]), or nonspecific polyclonal activation of memory cells (*27*). As a bias favoring stability, this overestimate of numbers of cases has limited consequences for surveillance purposes. However, for the individual patient, specificity of IgM in serum samples should be strongly considered.

The main conclusion of this study is that existing vaccination recommendations for travelers to areas endemic for hepatitis should be emphasized. Furthermore, immunization of travelers should be made more accessible to risk groups through information campaigns and removal of financial barriers (insurance payments for pretravel advice and vaccinations are not universal). If removal of these barriers to vaccination of all travelers is deemed unlikely, the general vaccination of children against HAV should be considered, given the high number of imported infections among children and the evidence of secondary autochthonous transmission.

Efforts to communicate recommendations to previously unaware population groups, especially, but not exclusively, persons with migration backgrounds, have the capacity to strongly reduce the number of HAV infections in Germany. However, as long as vaccination recommendations are applied only to travelers and overall immunity in the population remains low or decreases further, risk for secondary transmission of imported infections remains high.

## References

[1] Viral Hepatitis Prevention Board. Epidemiology of hepatitis A in Germany, 2004 [cited 2009 Jul 28]. Available from http://www.vhpb.org/files/html/Meetings_and_publications/Viral_Hepatitis_Newsletters/vhv12n3.pdf

[2] Thierfelder W, Hellenbrand W, Meisel H, Schreier E, Dortschy R. Prevalence of markers for hepatitis A, B and C in the German population. Results of the German National Health Interview and Examination Survey 1998. Eur J Epidemiol. 2001;17:429–35. 10.1023/A:1013792013184 11855576

[3] Swedish Institute for Infectious Disease Control. Data and statistics: hepatitis A; 2008 [cited 2008 Dec 19]. Available from http://www.smittskyddsinstitutet.se/in-english/statistics/hepatitis-a

[4] Robert Koch-Institut. Hepatitis A. Infektionsepidemiologisches Jahrbuch für 2007. Berlin: The Institut; 2008.

[5] Caractéristiques et expositions à risque des cas notifies d’hépatite aiguë A par classe d’âge, France. 2006–2007 [cited 2009 Jan 20]. Available from http://www.invs.sante.fr/surveillance/hepatite_a

[6] Nainan OV, Xia G, Vaughan G, Margolis HS. Diagnosis of hepatitis A virus infection: a molecular approach. Clin Microbiol Rev. 2006;19:63–79. 10.1128/CMR.19.1.63-79.2006 16418523 PMC1360271

[7] Thompson JD, Higgins DG, Gibson TJ. CLUSTAL W: improving the sensitivity of progressive multiple sequence alignment through sequence weighting, position-specific gap penalties and weight matrix choice. Nucleic Acids Res. 1994;22:4673–80. 10.1093/nar/22.22.4673 7984417 PMC308517

[8] Tamura K, Dudley J, Nei M, Kumar S. MEGA4: Molecular Evolutionary Genetics Analysis (MEGA) Software Version 4.0. Mol Biol Evol. 2007;24:1596–9. 10.1093/molbev/msm092 17488738

[9] German Statistical Office. Population with a migration background: results of the microcensus 2006 Wiesbaden (Germany): The Office; 2008.

[10] Nielsen US, Larsen CS, Howitz M, Petersen E. Hepatitis A among Danish travellers 1980–2007. J Infect. 2009;58:47–52. 10.1016/j.jinf.2008.10.010 19059649

[11] Angell SY, Cetron MS. Health disparities among travelers visiting friends and relatives abroad. Ann Intern Med. 2005;142:67–72. 15630110 10.7326/0003-4819-142-1-200501040-00013

[12] Ceyhan M, Yildirim I, Kurt N, Uysal G, Dikici B, Ecevit C, Differences in hepatitis A seroprevalence among geographical regions in Turkey: a need for regional vaccination recommendations. J Viral Hepat. 2008;15(Suppl 2):69–72. 10.1111/j.1365-2893.2008.01034.x 18837839

[13] Mutsch M, Spicher VM, Gut C, Steffen R. Hepatitis A virus infections in travelers, 1988–2004. Clin Infect Dis. 2006;42:490–7. 10.1086/499816 16421793

[14] Van Herck K, Van Damme P, Castelli F, Zuckerman J, Nothdurft H, Dahlgren AL, Knowledge, attitudes and practices in travel-related infectious diseases: the European airport survey. J Travel Med. 2004;11:3–8. 14769280 10.2310/7060.2004.13609

[15] Zwar N, Streeton CL. Pretravel advice and hepatitis A immunization among Australian travelers. J Travel Med. 2007;14:31–6. 10.1111/j.1708-8305.2006.00088.x 17241251

[16] Gervelmeyer A, Nielsen MS, Frey LC, Sckerl H, Damberg E, Molbak K. An outbreak of hepatitis A among children and adults in Denmark, August 2002 to February 2003. Epidemiol Infect. 2006;134:485–91. 10.1017/S0950268805005200 16194292 PMC2870412

[17] Hoebe CJ. Hepatitis A epidemic in Heerlen in late 1996, importance of immunization in immigrant children [in Dutch]. Ned Tijdschr Geneeskd. 1998;142:521–5. 9623099

[18] Sonder GJ, Bovee LP, Baayen TD, Coutinho RA, van den Hoek JA. Effectiveness of a hepatitis A vaccination program for migrant children in Amsterdam, The Netherlands (1992–2004). Vaccine. 2006;24:4962–8. 10.1016/j.vaccine.2006.03.075 16675076

[19] van Steenbergen JE, Tjon G, van den Hoek A, Koek A, Coutinho RA, Bruisten SM. Two years’ prospective collection of molecular and epidemiological data shows limited spread of hepatitis A virus outside risk groups in Amsterdam, 2000–2002. J Infect Dis. 2004;189:471–82. 10.1086/381152 14745705

[20] Robertson BH, Jansen RW, Khanna B, Totsuka A, Nainan OV, Siegl G, Genetic relatedness of hepatitis A virus strains recovered from different geographical regions. J Gen Virol. 1992;73:1365–77. 10.1099/0022-1317-73-6-1365 1318940

[21] Robert Koch Institut. Hepatitis A: zeitgleiche Ausbrüche in zwei benachbarten Landkreisen in Hessen und Rheinland-Pfalz. Epidemiologisches Bulletin. 2006;12:147–9.

[22] Schenkel K, Bremer V, Grabe C, Van Treeck U, Schreier E, Hohne M, Outbreak of hepatitis A in two federal states of Germany: bakery products as vehicle of infection. Epidemiol Infect. 2006;134:1292–8. 10.1017/S0950268806006212 16650329 PMC2870508

[23] Robert Koch Institut. Hepatitis A: zu einer aktuellen Häufung in München. Epidemiologisches Bulletin. 2003;29:223–4.

[24] Frank C, Walter J, Muehlen M, Jansen A, van Treeck U, Hauri AM, Major outbreak of hepatitis A associated with orange juice among tourists, Egypt, 2004. Emerg Infect Dis. 2007;13:156–8. 10.3201/eid1301.060487 17370535 PMC2725821

[25] Kao HW, Ashcavai M, Redeker AG. The persistence of hepatitis A IgM antibody after acute clinical hepatitis A. Hepatology. 1984;4:933–6. 10.1002/hep.1840040525 6090293

[26] Fikar CR, McKee C. False positivity of IgM antibody to Epstein-Barr viral capsid antigen during acute hepatitis A infection. Pediatr Infect Dis J. 1994;13:413–4. 10.1097/00006454-199405000-00016 8072826

[27] Roque-Afonso AM, Grangeot-Keros L, Roquebert B, Desbois D, Poveda JD, Mackiewicz V, Diagnostic relevance of immunoglobulin G avidity for hepatitis A virus. J Clin Microbiol. 2004;42:5121–4. 10.1128/JCM.42.11.5121-5124.2004 15528704 PMC525178

